# Psychometric properties of the stagnation scale in medication overuse headache patients

**DOI:** 10.1186/1129-2377-16-2

**Published:** 2015-02-19

**Authors:** Marco Innamorati, Maurizio Pompili, Denise Erbuto, Federica Ricci, Monica Migliorati, Dorian A Lamis, Mario Amore, Paolo Girardi, Paolo Martelletti

**Affiliations:** Department of Neurosciences, Mental Health and Sensory Organs, Suicide Prevention Center, Sant’Andrea Hospital, Sapienza University of Rome, Rome, Italy; Department of Psychiatry and Behavioral Sciences, Emory University School of Medicine, Atlanta, GA USA; Department of Neurosciences, Rehabilitation, Ophthalmology and Genetics, Section of Psychiatry, University of Genova, Genova, Italy; Department of Clinical and Molecular Medicine, Sapienza University of Rome and Regional Referral Headache Centre, Sant’Andrea Hospital, Rome, Italy

**Keywords:** Medication overuse headache, Stagnation, Depression, Structural equation modeling

## Abstract

**Background:**

Medication-overuse headache (MOH) is often comorbid with emotional disturbances, contributing to poorer outcomes. The aims of the present study were to assess the psychometric properties of the Stagnation Scale in a sample of MOH patients, and to compare two factor models: a three-factor model reported in previous studies and a proposed bi-factor model.

**Methods:**

Consecutive adult outpatients (*N* = 310) admitted to the Regional Referral Headache Centre of the Sant’Andrea Hospital in Rome (Italy) were administered the Stagnation Scale and two questionnaires measuring depression and perceived disability.

**Results:**

The original three-factor model demonstrated an adequate fit to the data (*χ*^*2*^_101_ = 238.70; *p* < 0.001; Root Mean Square Error of Approximation [RMSEA] = 0.07; 90% CI of RMSEA = 0.06 / 0.08; Comparative Fit Index [CFI] = 0.98; Weighted Root Mean Square Residual [WRMR] = 0.75). However, the bi-factor model had a comparable or even better fit, with a RMSEA of 0.05 (90% CI: 0.04 / 0.07), providing strong evidence for an absolute fit to the data (*χ*^*2*^_88_ = 161.43; *p* < 0.001; RMSEA = 0.05; 90% CI of RMSEA = 0.04 / 0.07; CFI = 0.99; WRMR = 0.56). The stagnation general factor and all the group factors correlated significantly and positively with convergent measures.

**Conclusions:**

There is support for the use of the Stagnation Scale in MOH patients, with the goal of better understanding the role of psychological factors in the evolution and course of the disorder.

## Background

Medication-overuse headache (MOH) is characterized by headache attacks occurring 15 or more days per month for at least 3 months and is associated with the intake of a medication (e.g., ergotamine, triptan, analgesic) on ≥10 days/month on a regular basis for ≥3 months [1, 2]. Its prevalence in the adult general population is estimated to be 1-2% [3–8]. MOH may vary between around 4% [9] and 8% in headache patients referred to neurological clinics [10], and may be diagnosed in up to 29% of patients from tertiary referral centers for chronic pain [11].

MOH is often comorbid with emotional disturbances and disordered personality traits [12, 13], which seem to play a role in the evolution of migraine to MOH and are associated with poorer outcomes [14]. Psychological disturbances may also be risk factors for a later development of MOH; for example, a prospective 11-year longitudinal study demonstrated that anxiety and depression with musculoskeletal and gastrointestinal complaints were risk factors for MOH, but not for chronic headache (i.e., a form of headache with attacks occurring 15 or more days per month for at least 3 months) without medication overuse [15]. The presence of psychopathology has also been used to distinguish complicated cases of MOH (MOH Type II) [16–18]. Thus, it is important to use instruments that can identify a variety of psychiatric disorders, emotional disturbances, and disordered personality traits among patients with MOH who are refractory to usual care [19]. Nevertheless, Weeks and Weier [20] pointed out that there is no agreement as to which tests are the most effective or the most promising in identifying psychological factors in headache patients. In a review of screening tools for psychiatric comorbidity in headache patients, Maizels et al. [19] recommend screening all headache patients at least for anxiety and depression. Moreover, the authors suggested more comprehensive multidimensional psychiatric screening when patients respond poorly to standard headache management. They also suggested the use of some specific instruments such as the Beck Depression Inventory [21], Beck Anxiety Inventory [22], Patient Health Questionnaire [23], and Mini-International Neuropsychiatric Interview [24].

Recently, our group [25] investigated the role of *stagnation*, an old diagnostic entity of traditional Chinese medicine, in a sample of 69 patients with chronic migraine to better understand the beliefs that patients may have regarding their autonomy/disability. Our results indicated that stagnation was associated with higher perceived disability independent of the severity of depression, and that it could be useful for predicting perceived disability among patients with chronic migraine. This pilot study was also the first investigation assessing the utility of the concept of stagnation in western patients with chronic headache. According to the traditional Chinese medicine, the stagnation syndrome is characterized by a cluster of mind/body obstruction-like symptoms such as feeling that something is stuck in the throat, chest and stomach. The cause of stagnation is hypothesized to be the repression of emotions [26]. If emotions are repressed for a long time, this can lead to a number of mind/body dysfunctions, such as suppressed emotions, especially anger, sleeping disturbances, dizziness, fatigue, a feeling of obstruction in swallowing, indigestion and bowel dysfunctions [27]. The hypothesis that the repression of emotions may lead to headache is also present in western psychological theories [28, 29]. Recently, Sances and colleagues [14] investigated personality factors associated with negative outcomes in MOH patients during a 3-year follow-up study. Utilizing the Minnesota Multiphasic Personality Inventory, 2^nd^ edition (MMPI-2) [30], the authors reported that those who never stopped their drug overuse, compared to other patients, had higher scores on several dimensions of the MMPI-2, including hypochondriasis (individuals with high scores on this scale report the presence of many somatization-like symptoms), repression (individuals with high scores on this scale report the use of repression, denial, and rationalization, and the lacking of self-insight, unwillingness to discuss personal shortcomings, they usually appear over-controlled), and overcontrolled hostility (individuals with high scores on this scale report a tendency to use denial and repression of aggressive impulses). Nicholson et al. [31], while evaluating whether anger and anger expression are different between individuals with and without headache after controlling for depression and anxiety, reported that headache patients have higher levels of anger-in (higher levels of anger-in are indicative of a failure in expressing anger), even after controlling for other variables. Headache and migraine have also been demonstrated to be strong and independent predictors of the presence of somatic symptoms in patients with major depression. Furthermore, somatosensory amplification (i.e., the tendency to perceive normal bodily sensations as unusually intense and disturbing) has been shown to be associated with the number of attacks and disability in a small group of migraine patients [32].

Ng et al. [27, 33–35] operationalized the construct of stagnation through the development of a new scale: the Stagnation Scale. In their initial study, the authors administered the Stagnation Scale, the Beck Depression Inventory [36], and the General Health Questionnaire (GHQ-12) [37] to a sample of 602 Chinese adults. Stagnation scores were moderately associated with depression, while a different pattern of associations between depression and demographic variables emerged suggesting a distinct clinical syndrome [27]. More recently, the authors conducted a confirmatory factor analysis (CFA) study with a random community sample of 755 adults recruited in Hong Kong [35]. The fit indices for a three-factor model reported in the previous studies were not completely satisfactory (comparative fit index [CFI] = 0.94, root mean square error of approximation [RMSEA] = 0.09, standardized root mean square residual [SRMR] = 0.04); however, when the authors allowed residual covariance between items 15 and 16, the resultant model fit improved and was deemed adequate (CFI = 0.95; RMSEA = 0.08 [90% CI of RMSEA = 0.07 / 0.08]; SRMR = 0.04) [35]. The three dimensions are Overattachment, Body–Mind Obstruction, and Affect–Posture Inhibition [27, 33, 34]. Items included in the Overattachment subscale investigate preoccupation or fear of losing what one possesses, being not as accomplished as others, and/or being unable to let go of some matters. In Body–mind Obstruction, the theme is somatic obstruction-like symptoms such as a feeling that the stomach is clogged and that something is obstructing the throat. In Affect–Posture inhibition, the themes are being overly self-conscious, heightened awareness and uneasiness, resulting in inhibited facial expression of affect and postural movement.

Correlational analyses with concurrent measures also indicated that the Stagnation Scale was moderately associated with the Physical Distress subscale of the Body–Mind–Spirit Well-Being Inventory (*r* = 0.59) [38], and strongly related to the GHQ-12 (*r* = 0.69) [37] and the anxiety and depression scores on the Hospital Anxiety and Depression Scale (HADS; *r* = 0.76, 0.60, respectively for the anxiety and the depression subscales) [35, 39]. Finally, using participants’ subjective appraisal of illness condition as the criterion, the authors carried out a receiver operating characteristic (ROC) curve analysis to evaluate the optimal cutoffs on the stagnation score with an aim of balancing false-positive and false-negative rates in predicting illness condition by self-appraisal. The researchers reported that the optimally balanced cutoff points on the Stagnation Scale were 50 for the total score (false-positive and -negative rates, 0.26 and 0.23, respectively), 27 on the Overattachment subscale (false-positive and -negative rates, 0.22 and 0.23), 14 on the Body–Mind Obstruction subscale (false-positive and -negative rates, 0.21 and 0.23), and 10 on the Affect–Posture Inhibition (false-positive and -negative rates, 0.24 and 0.26) [35].

Given that our previous research has supported the utility of the concept of stagnation when investigating the psychological health of patients with chronic headache, the psychometric properties of the Stagnation Scale have not been evaluated in western patients. Thus, the aim of the present study was to assess the psychometric properties of an Italian version of the Stagnation Scale in a sample of MOH patients. First, we assessed dimensionality of the Stagnation Scale by means of structural equation modeling (SEM). Specifically we compared a three-factor model reported in previous studies, with a proposed bi-factor model [40]. In the bi-factor model, each item loads on a general factor and a group factor [41]. The general factor explains the covariance shared by all items. The group factors account for the covariance independent from the general factor. The general factor and group factors are uncorrelated and account for the covariance simultaneously and independently for each item. The advantage of the bi-factor model over the original three-factor model is that it hypothesizes the existence of a latent trait and the quasi-unidimensionality of the questionnaire. In fact, although stagnation may manifest itself with psychological, behavioral, and physiological symptoms, it was theorized as a single factor despite the heterogeneous syndrome with a common cause of repression of emotions. Moreover, although reporting the validity of a three-factor model, the authors of the Stagnation Scale calculated a total score consisting of the sum of the three factor scores. Thus, a bi-factor model with the presence of a general factor may conform to the theory better than a structure with 3 first-order factors, especially if all items load more strongly on the general factor than on the group factors. Second, we investigated whether there were sex differences on the Stagnation Scale scores at the item and scale levels. Previously, Ng et al. [27], reported sex differences in the Body–Mind Obstruction factor with women reporting higher mean scores, but not in the total and the other dimension scores. Third, we investigated reliability and convergent validity with depression severity and a measure assessing patient beliefs about the negative effects the illness has on his/her health and daily activities. We hypothesized that the Stagnation Scale dimensions would have moderate to strong correlations with these convergent measures. Lastly, considering Meng, Rosenthal and Rubin’s [42] approach, we hypothesized that the general factor would exhibit discriminant validity in its associations with depression and negative beliefs about the impact of the illness when compared to group factors.

## Methods

### Sample and procedures

Participants were 310 adult outpatients (189 men, 121 women) consecutively admitted to the Regional Referral Headache Centre of the Sant’Andrea Hospital in Rome, between September 2011 and December 2013. Inclusion criteria were a diagnosis of MOH, and an age of 18 years or older. Exclusion criteria were comorbidity with major disorders of the central nervous system (e.g., Parkinson disease, dementia, epilepsy), delirium and/or any condition affecting the patient’s ability to complete the assessment, including refusal of informed consent. The average age of participants was 48.50 years (Min./Max.: 20/88; *SD* = 12.44). Other characteristics of the sample are reported in Table 1. Patients participated voluntarily in the study, and each subject provided written informed consent. The study protocol received ethics approval from the local research ethics review board.

**Table 1:** **Sociodemographic and clinical characteristics of the sample (** ***N*** **= 310)**

	Count	Percentage
Men	189	61.0%
Age – Mean (Standard deviation)	48.50	(12.44)
Marital status		
Married	205	66.2%
Single	67	21.7%
Widowed	11	3.5%
Divorced	27	8.6%
School attendance ≤ 8 years	103	33.2%
Job		
Employed	255	82.2
Unemployed	34	10.9%
Retired	21	6.9%
Migraine without aura	310	100%
Migraine with aura	9	2.9%
Headache onset		
Preschool	74	24.0%
Puberty	119	38.3%
Adulthood	117	37.8%
Frequency of attacks: daily	161	52.0%
Familiarity for headache	215	69.5%

### Measures

Patients were administered the Stagnation Scale, the Beck Depression Inventory – II (BDI-II) [21], and the Italian Perceived Disability Scale (IPDS) [43].

The Stagnation Scale is a 16-item questionnaire assessing stagnation syndrome (items are listed in Table 2). Respondents were asked to rate each item on a ten-point anchored numeric scale (“1” indicating “has not occurred at all” and “10” indicating “occurring every single moment”), according to how they felt in the last two weeks. In previous research, the Cronbach’s alpha for the full scale was 0.91 [27], and the three subscales had Cronbach’s alphas ranging between 0.82 and 0.88. The composite stagnation scale has shown significant positive correlations with a self-report measure of depression [27].

**Table 2:** **Standardized factor loadings (standard errors) for the alternative models (upper: bi-factor model; lower: three-factor model)**

Items	Overattachment	Body-Mind obstruction	Affect-Posture inhibition	Stagnation
*Item 1-* I fear losing what I possess.	0.38* (0.06)	-	-	0.62* (0.04)
	0.72* (0.03)			
*Item 2-* I get obsessed with details about certain matters.	0.45* (0.05)	-	-	0.64* (0.04)
	0.77* (0.03)			
*Item 3-* It concerns me that I am not as accomplished as others.	0.40* (0.06)	-	-	0.60* (0.04)
	0.71* (0.03)			
*Item 4-* Thinking about troubling things uses up a lot of my energy.	0.44* (0.04)	-	-	0.70* (0.03)
	0.82* (0.03)			
*Item 5-* I get overly attached to some matters, unable to let go.	0.49* (0.04)	-	-	0.71* (0.04)
	0.85* (0.03)			
*Item 6-* I moan and sigh.	0.20* (0.06)	-	-	0.64* (0.04)
	0.69* (0.04)			
*Item 7-* I still miss the things I have already lost.	0.22* (0.06)	-	-	0.62* (0.04)
	0.67* (0.04)			
*Item 8-* I feel as if my stomach is clogged.	-	0.36* (0.06)	-	0.77 (0.03)
		0.83* (0.02)		
*Item 9-* I feel as if there is something obstructing my throat.	-	0.48* (0.06)	-	0.76* (0.04)
		0.84* (0.03)		
*Item 10-* I feel my heart beating disquietly.	-	0.40* (0.06)	-	0.71* (0.03)
		0.78* (0.03)		
*Item 11-* My head feels heavy and dizzy.	-	0.08 (0.07)	-	0.68* (0.04)
		0.70* (0.04)		
*Item 12-* I have an indescribable fear inside.	-	-0.01 (0.06)	-	0.84* (0.03)
		0.85* (0.03)		
*Item 13-* My gait is constrained.	-	-	0.21* (0.06)	0.78* (0.04)
			0.85* (0.03)	
*Item 14-* My sitting posture is rigid.	-	-	0.38* (0.07)	0.67* (0.04)
			0.78* (0.03)	
*Item 15-* My facial expressions are unnatural.	-	-	0.62* (0.06)	0.72* (0.04)
			0.87* (0.02)	
*Item 16-* My facial expressions are flat.	-	-	0.42* (0.06)	0.72* (0.04)
			0.84* (0.03)	

*p < 0.001.

We translated and adapted the Italian version of the Stagnation Scale from an English version provided from the authors of the measure. One author (MI) translated the original English version in Italian and a second researcher (MP) blindly back-translated the measure to the source language. The back-translated version was submitted to the authors of the Stagnation Scale, who suggested slight word changes to four items. After having improved the Italian version of the questionnaire and revised the back-translated version, we resubmitted it to the authors of the original version of the scale who found no discrepancies between our version and the original measure. The definitive Italian version of the questionnaire was administered to a small sample of 5 patients (2 women and 3 men; age 52.80 ± 15.87; range 30/70 years) with chronic headache who participated in a previous study [25]. They evaluated items and instructions for their perceived comprehensibility using a four point Likert scale (extremely easy to understand, quite easy to understand, quite hard to understand, extremely hard to understand). All the items and the instructions were rated as extremely easy or quite easy to understand from patients.

The IPDS measures people’s beliefs regarding autonomy/disability and the negative impact the illness has on their health and daily activities in different situations of life (e.g., “my body is weak and unreliable”, “I will have to worry about my health conditions all my life long”; “I boil over more easier than in the past”). It is composed of 20 items rated on a 5-point Likert scale (from completely false to completely true). The raw scores range from 0 to 80. In a previous study, the IPDS demonstrated good reliability in a sample of patients with chronic daily headache [43].

The BDI-II is a 21-item self-report inventory designed to assess the presence and severity of depressive symptoms according to the DSM-IV criteria. Respondents have to endorse specific statements that reflect their feelings over the last two weeks. Each statement is rated on a 4-point Likert scale ranging from 0 to 3 on the basis of symptom severity, which yields a summed minimum score of 0 and a maximum score (indicative of high depressive symptomology) of 63. An example of an item on the BDI-II is “Sadness”, with response options being 0 (I do not feel sad), 1 (I feel sad much of the time), 2 (I am sad all of the time), and 3 (I am so sad or unhappy that I can’t stand it). Good estimates of internal consistency and concurrent validity have been demonstrated for the Italian version of the BDI-II [44, 45].

### Statistical analysis

To test the fit of the three-factor and the bi-factor models, the data were subjected to a confirmatory factor analysis (CFA) by means of Mplus 6.0 [46]. The extraction of the factors was conducted employing the Mean- and Variance-adjusted Weighted Least Square (WLSMV) estimation method on a polychoric correlation matrix. Model fit was assessed using the following indices: 1) the RMSEA, a measure of absolute fit. Values between 0.05 and 0.08 are indicative of good adequacy of the model, and below 0.05 deemed strong evidence of absolute fit [47, 48]; 2) the CFI. Values greater than 0.95 / 0.96 for these indicators are indicative of good model fit; 3) the Weighted Root Mean Square Residual (WRMR): Yu [49] recommend that a model with a WRMR of less than 1.0 indicates good fit; and 4) the chi-squared (*χ*^*2*^) test. *P*-values greater than 0.05 indicate that the model is adequate to the data, although the *χ*^*2*^ test over-reject true models for large samples or under non-normality [49]. Considering limitations in the use of *χ*^*2*^ statistic, we also reported the relative *χ*^*2*^ (*χ*^*2*^/degree of freedom) statistic [50]. The relative *χ*^*2*^ should be less than 2 for well-fitting models [51], although diverse values have also been proposed [50, 52]. We did not use any specific test to compare the two models. Although some statistics have been proposed for comparing the fit and parsimony of non-nested models (e.g., the Akaike information criterion, the consistent version of the Akaike Information Criterion, the Bayesian Information Criterion, the Expected Cross-Validation Index, or the Vuong’s Likelihood Ratio Test) [53–56], they all require the use of the maximum likelihood estimator; however they cannot be computed when using weighted least squares estimators as we did in this study.

For each item, we reported the standardized lambda coefficients (*λ*) expressing the relationships between the factor and its observed variables (used to measure the validity of the indicator; that is, how well they measure the latent dimension). As measures of reliability, we reported Cronbach’s alpha (*α*), McDonald's omega, and inter-item mean indices of correlation (*r*_*ii*_) for tests of homogeneity. McDonald's omega can be interpreted as the square of the correlation between the scale score and the latent variable common to all the indicators in the infinite universe of indicators of which the scale indicators are a subset [57]. McDonald's omega coefficients were calculated with statistical software Factor 9.2 [58]. One-way Fisher exact tests and t-tests were used to calculate significance of differences between sex. Convergent validity with the IPDS and the BDI-II was evaluated by using Pearson’s *r* indices of correlations. We used the approach recommended by Meng, Rosenthal and Rubin [42] to examine discriminant validity of the general and group factors of the Stagnation Scale with measures of depression and perceived disability. This procedure involves performing a Fisher Z transformation on the correlation coefficients so that they can be compared via a *t*-test.

## Results

Eighteen patients failed to answer one or more items of the Stagnation Scale and their data were not included in the structural equation models. An average of 8 patients (*SD* = 1) failed to complete each one of the seventeen items with scarce variation among items (range: 7/10 items). A significant difference between sex was evident (t_301_ = 18.20; *p* < 0.001) with male patients reporting more non completed items (4.82 ± 0.88 vs. 3.18 ± 0.53) as compared to their female counterparts. When comparing sex differences for items mean scores, no significant differences were found (not reported in the tables).

### Model fit

Fit statistics for the alternative SEM models are reported in Table 3. The analyses demonstrated that the original three-factor model had an adequate fit to the data. However, the bi-factor model had a comparable or even better fit, with a RMSEA of 0.05 (90% CI: 0.04 / 0.07) indicating strong evidence of absolute fit to the data. When inspecting factor loadings of the three-factor model (Table 2), all of the items significantly loaded on the hypothesized dimension; whereas, when examining factor loadings of the bi-factor model, all the items more strongly loaded on the general factor than they did on the group factors. Only two items (items 11 and 12) did not load significantly on the hypothesized group dimensions (both items were hypothesized to load on the Body-Mind Obstruction dimension), although they significantly loaded on the general factor.

**Table 3:** **Fit statistics for the alternative models (Estimator: Mean- and Variance-adjusted Weighted Least Square)**

	Chi-Square (χ ^2^ )	Relative chi-square (χ ^2^ /df)	Root mean square error of approximation (RMSEA) (90%CI)	Comparative Fit Index (CFI)	WRMR (Weighted root mean square residual)	Degree of freedom (df)
Three-factor model	238.70*	2.36	0.07 (0.06/0.08)	0.98	0.75	101
Bi-factor model	161.43*	1.83	0.05 (0.04/0.07)	0.99	0.56	88

*Significant for *p* < 0.001.

### Psychometric properties of the stagnation scale

Reliabilities and descriptive statistics for all measures administered are reported in Table 4. Omega coefficients were 0.93 for the Stagnation Scale general factor score, 0.87 for Overattachment, 0.85 for Body-Mind Obstruction, and 0.83 for Affect-Posture Inhibition, indicating that around 93% of the variance of the Stagnation Scale scores was attributable to a latent factor common to all test items. Male and female patients did not differ for their mean scores on the stagnation general factor or any of the group dimensions (Table 4). The stagnation general factor score (BDI-II = 0.67; IPDS = 0.63; *r* coefficients significant for *p* < 0.001), and all the group dimensions (Overattachment: BDI-II = 0.61; IPDS = 0.56; Body-Mind Obstruction: BDI-II = 0.61; IPDS = 0.57; Affect-Posture Inhibition: BDI-II = 0.58; IPDS = 0.53; all coefficients significant at *p* < 0.001) were significantly and positively correlated with convergent measures of depression and IPDS scores. According to the approach recommended by Meng et al. [42], the general factor correlated more strongly (*p* < 0.01) with the convergent measures of depression and perceived disability than the group factors.

**Table 4:** **Descriptive statistics and reliability indices (** ***n*** **= 310)**

	***M (SD)***	Men ***n*** = 189	Women ***n*** = 121	t-test (DF = 309)	***P***	Cronbach alpha	Inter-item mean correlation
Stagnation total score	51.64 (29.70)	50.81 (28.66)	52.94 (31.34)	-0.61	0.54	0.93	0.44
Overattachment	24.78 (14.61)	24.15 (13.92)	25.79 (15.64)	-0.96	0.34	0.87	0.49
Body-Mind obstruction	16.61 (10.57)	16.66 (10.29)	16.54 (11.04)	0.09	0.93	0.85	0.53
Affect-Posture inhibition	10.36 (8.18)	10.21 (7.94)	10.61 (8.56)	-0.42	0.68	0.83	0.56
BDI-II	11.15 (9.43)	11.74 (9.65)	10.56 (9.98)	1.04	0.30	0.92	0.36
BDI-II ≥ 20	16.4%	16.6%	16.1%	-	0.52	-	-
IPDS	26.09 (16.63)	27.48 (16.76)	23.95 (16.27)	1.82	0.07	0.92	0.36

BDI-II = Beck Depression Inventory – II; IPDS = Italian Perceived Disability Scale.

## Discussion

Our results suggest that the three-factor structure reported in previous studies assessing the characteristics of the original version of the Stagnation Scale [27, 33–35] is adequate to represent the structure of the Italian version of the Stagnation Scale; however, the bi-factor model had comparable or even better fit to the data, and the RMSEA suggested strong evidence of absolute fit to the data of the latter model. This finding suggests that a general factor common to all the sixteen items of the Stagnation Scale and three specific latent factors underlying groups of items are simultaneously present. Furthermore, items loaded more strongly on the general factor than on the group factors and the McDonald's omega indicated that the general factor was able to capture a large part of the variance of the test scores. These results suggest that the structure of this instrument can be considered mostly unidimensional, and are in line with the theory which defines stagnation as a single yet heterogeneous syndrome manifesting itself with psychological, behavioral, and physiological symptoms. Thus, a bi-factor model with the presence of a general factor may be able to capture a large part of the variance of the test scores, and three group factors correlated only because the single general factor may conform well to the theory. Our results are also consistent with original authors of the Stagnation Scale who report a total score along with the three dimensions scores.

The Stagnation scores were strongly associated with depression severity and with patients’ beliefs about the negative effects the illness has on their health and daily activities. This is consistent with previous studies conducted in Chinese samples [27], although our sample demonstrated correlation coefficients greater than those reported in the older Chinese study. Specifically, the Chinese study authors reported correlation coefficients ranging from 0.37 to 0.55 between Stagnation scores and the Beck Depression Inventory [27], which were lower than those reported in the more recent study with a correlation coefficient of 0.60 between Stagnation scores and the HADS depression subscale [35]. Despite the strong association between depression severity and stagnation, the stagnation scale still has a large amount of unique variance and should be considered a construct independent from depression and anxiety, as indicated by the results of the study conducted by Ng et al. [35]. More importantly, stagnation scores are also strongly associated with negative beliefs about the effects of the illness on health and daily activities, and as demonstrated in our previous study [25], they were able to predict patients’ beliefs regarding one’s own perceived disability independent of depression severity.

Stagnation total scores showed a different pattern of correlations with depression and perceived disability when compared with group factors, indicating discriminant validity in its association with depression and perceived disability. Stagnation total scores correlated more strongly with convergent measures of depression and perceived disability than group factors, possibly because the group factors tap only one single aspect of the stagnation symptomatology, either psychological or physiological symptoms; whereas, it may be the coexistence of perceived psychological, behavioral, and physiological symptoms that is particularly burdensome for patients.

To date, numerous psychometric instruments have been used in studies involving headache patients; however, an agreement has not been reached as to which tests are most effective in identifying psychological factors in this population [20]. Although screening for depression and anxiety in headache patients in primary care settings is recommended, more comprehensive assessment of psychopathology and psychological factors is necessary among patients who respond poorly to standard headache management or with patients attending headache specialty settings [19]. The Stagnation Scale may be used to assess the presence of a cluster of somatization-like symptoms, and preoccupations and fears. The assessment of stagnation could be useful considering that past literature has reported that the presence of somatosensory amplification may be predictive of the number of attacks and disability in migraine patients [32] or negative beliefs that the impact the illness has on them [25]. Furthermore, considering its strong association with depression and the independency of this construct, the Stagnation Scale can be used to discriminate complex MOH cases (MOH Type II) characterized by the comorbidity with multiple psychiatric disorders and a long history of relapses [59]. Another strength of the Stagnation Scale is the fact that its properties have been evaluated in a large sample of chronic headache patients; whereas, other commonly used measures have been investigated with different populations. Nevertheless, we must also consider the fact that the stagnation syndrome is not part of the western tradition, and this construct has not been previously examined among western patients or in the general population, so our results may not be generalizable to other populations. For example, the high presence of male patients in our sample may have had an impact on our results, although male and female patients did not differ in their scores on the Stagnation Scale. Future studies with larger samples should investigate the invariance of the structure between sex groups.

### Strengths and limitations of the study

We have to consider our study results in light of some limitations. For example, we only used self-reported measures which could have been biased by social desirability [60, 61], and our results are not generalizable to patients with other diagnoses. Furthermore, although we evaluated the association between stagnation and depression, we did not examine whether stagnation was also associated with anxiety or other forms of psychopathology. For instance, we did not assess the presence of comorbid mood or anxiety disorders, although previous research has suggested that psychiatric disorders may be present in a high percentage of patients [62–64]. Nonetheless, to our knowledge, this is the first study to evaluate the psychometric properties of the Stagnation Scale in a large western sample of MOH patients. However, future research is needed and should focus on the study of other psychometric properties of the Stagnation Scale (e.g., test-retest reliability and predictive validity).

## Conclusion

We found support for the use of the Italian version of the Stagnation Scale in patients with MOH, with the goal of better understanding the role of psychological factors on the evolution and course of the disorder. In fact, MOH patients reported clusters of mind/body obstruction-like symptoms, such as feeling something stuck in the throat, chest and stomach, which are correlated with the perception of one’s own disability.
